# Do direct oral anticoagulants (DOACs) cause delayed surgery, longer length of hospital stay, and poorer outcome for hip fracture patients?

**DOI:** 10.1007/s41999-020-00319-w

**Published:** 2020-07-23

**Authors:** Sunniva Leer-Salvesen, Eva Dybvik, Anette H. Ranhoff, Bjørn Liljestrand Husebø, Ola E. Dahl, Lars B. Engesæter, Jan-Erik Gjertsen

**Affiliations:** 1 Department of Clinical Medicine, University of Bergen, Bergen, Norway; 2 The Norwegian Hip Fracture Register, Department of Orthopaedic Surgery, Haukeland University Hospital, Bergen, Norway; 3 Department of Clinical Science, University of Bergen, Bergen, Norway; 4 Department of Chronic Diseases and Aging, Norwegian Institute of Public Health, Oslo, Norway; 5 Diakonhjemmet Hospital, Oslo, Norway; 6 Department of Anaesthesia, Haukeland University Hospital, Bergen, Norway; 7 Innlandet Hospital Trust, Elverum, Norway; 8 Thrombosis Research Institute, London, UK

**Keywords:** Hip fracture, Orthogeriatrics, Surgical delay, Anaesthesia, Direct oral anticoagulants (DOAC), New oral anticoagulants (NOAC)

## Abstract

**Purpose:**

The perioperative consequences of direct oral anticoagulants (DOACs) in hip fracture patients are not sufficiently investigated. The primary aim of this study was to determine whether DOAC-users have delayed surgery compared to non-users. Secondarily, we studied whether length of hospital stay, mortality, reoperations and bleeding complications were influenced by the use of DOAC.

**Methods:**

The medical records of 314 patients operated for a hip fracture between 2016 and 2017 in a single trauma center were assessed. Patients aged < 60 and patients using other forms of anticoagulation than DOACs were excluded. Patients were followed from admission to 6 months postoperatively. Surgical delay was defined as time from admission to surgery. Secondary outcomes included length of hospital stay, transfusion rates, perioperative bleeding loss, postoperative wound ooze, mortality and risk of reoperation. The use of general versus neuraxial anaesthesia was registered. Continuous outcomes were analysed using Students *t* test, while categorical outcomes were expressed by Odds ratios.

**Results:**

47 hip fracture patients (15%) were using DOACs. No difference in surgical delay (29 vs 26 h, *p* = 0.26) or length of hospital stay (6.6 vs 6.1 days, *p* = 0.34) were found between DOAC-users and non-users. DOAC-users operated with neuraxial anaesthesia had longer surgical delay compared to DOAC-users operated with general anaesthesia (35 h vs 22 h, *p* < 0.001). Perioperative blood loss, transfusion rate, risk of bleeding complications and mortality were similar between groups.

**Conclusion:**

Hip fracture patients using DOAC did not have increased surgical delay, length of stay or risk of reported bleeding complications than patients without anticoagulation prior to surgery. The increased surgical delay found for DOAC-users operated with neuraxial anaesthesia should be interpreted with caution.

## Key summary points

**Aim:**

The aim of this study was to determine whether DOAC-users with a hip fracture have delayed surgery, longer length of hospital stay or altered risk of bleeding complications compared to non-users.

**Findings:**

DOAC-users with a hip fracture did not have increased surgical delay, length of stay or risk of reported bleeding complications compared to patients without anticoagulation prior to surgery.

**Message:**

Our study does not support delayed surgery for DOAC-users suffering a hip fracture.

## Introduction

The use of direct oral anticoagulants (DOACs) have emerged based on randomized clinical trials, active marketing and less demands concerning monitoring compared to warfarin. From 2014 to 2018, the prevalence of DOAC-users increased with 150% in Norway and the drugs as a group have surpassed warfarin [1]. Increasing use of DOACs has also been observed in Germany, Belgium and The Netherlands [2]. Suffering a hip fracture results in an evident excess mortality [3], and knowledge on how to reduce complications is, therefore, important. Reduced kidney function, co-medication, drug interaction and altered distribution may affect the clinical outcome in hip fracture patients using such anticoagulant compounds [4].

Systemic thromboembolic events are important causes of mortality [5, 6]. On the other hand, DOACs may accentuate bleeding triggered by trauma and surgery. Whether DOACs should be temporarily paused to avoid surgical and anaesthesiological complications and, if so, when it should be paused remains to be established. Anticoagulation has in several studies been identified as a risk factor for delayed hip fracture surgery [7–10]. Most guidelines advocate that hip fracture surgery should be performed within 48 h after admission, preferably within 24 h, to reduce the rate of medical complications and mortality [11–13]. Earlier studies have indicated that patients exposed for DOAC before the hip fracture wait longer for surgery than recommended in treatment guidelines [14–16]. The consequences of DOAC on semi-urgent surgery such as for hip fracture patients has not been thoroughly investigated.

Currently, there is need for guidelines on how to handle DOACs in the treatment of hip fracture patients. The primary aim of this study was to determine whether hip fracture patients using DOACs prior to the fracture have delayed surgery or longer length of hospital stay compared to non-DOAC-users. Secondarily, we wanted to investigate whether mortality and perioperative complications occur more frequently among hip fracture patients using DOAC.

## Methods

### Study design

This is a retrospective descriptive study of hip fracture patients operated at one Norwegian single trauma center December 2016–December 2017. We extracted 360 patients electronically from the hospital database using ICD-10 diagnosis codes S72.0–S72.2. Demographic data and surgical outcomes for the included patients were retrieved directly from patient records by one experienced researcher (SLS). Patient records at the hospital consisted of day-to-day documentation by the anaesthetists and orthopaedic surgeons and medical records logged by physicians and nurses. The Regional Ethics Committee (REK) classified the study as quality assurance, thus we did not need ethical assessment (case number 1366/REK). The hospital data protection officer approved the study.

### Patients

Patients with acute intracapsular or extracapsular hip fractures undergoing any type of surgery were included in the study. We aimed to compare hip fracture patients using DOAC at time of fracture with patients without anticoagulation at time of fracture. Patients under the age of 60 (*n* = 23) and patients using other forms of anticoagulation than DOACs (*n* = 23) were excluded, resulting in a study population of 314 patients.

### Outcomes

We stratified the patients according to the American Society of Anesthesiologists (ASA) classes 1–2 and 3–5 to compare comorbidity between the studied groups. When comparing the rate of cognitive impairment reported between the study groups, patients with unknown preoperative cognitive status were excluded (*n* = 20). Time from admission to surgery (surgical delay) was reported in hours and length of stay (LOS) in days. In-hospital mortality and both mortality and readmissions within 30 days and within 6 months of operation were registered. Blood transfusion rates and transfusion amounts (allogenic red blood cells infused in standardized units) were collected from the medical records signed by the responsible physicians. In-hospital guidelines recommended blood transfusion therapy to be administered for patients with a haemoglobin below 9 g/dL monitored at the wards. The concentration of haemoglobin was listed at admission and the morning after surgery and the difference was calculated (change in haemoglobin concentration). Intraoperative blood loss estimated by the surgical team was registered from the anaesthesia journal in milliliters (mL). Postoperative bleeding and wound complications were recorded if the intraoperative or postoperative journals by the physicians reported so. Wound ooze was defined as clinically identified ooze with or without bleeding described by the doctors postoperatively*.* The type of anaesthesia was registered as general anaesthesia (total intravenous anaesthesia (TIVA) or inhalational anaesthesia) or neuraxial anaesthesia (spinal anaesthesia). We compared surgical delay and LOS within the groups receiving neuraxial versus general anaesthesia.

### Statistical analysis

Our main outcome, surgical delay, was used to calculate the number of patients needed to achieve statistical significance between the groups. Based on guidelines from the Norwegian Knowledge Center hip fracture patients should preferable be operated within 24 h and no later than 48 h after admission [12]. Standard deviation was calculated from hip fracture patients with a surgical delay of less than 96 h reported to the Norwegian Hip Fracture Register and found to be 15.1 h. Based on alpha of 0.05 and beta of 0.9, 28 patients were needed in each group. Since 9.4% of Norwegian patients > 60 years were using DOAC in 2017 (Norwegian Institute of Public Health 2019), the total sample size was calculated to be 300. To account for exclusion criteria’s and missing information, we increased the sample size with 20%.

We performed univariate exploration of study variables; for continuous data, the assumption of homogeneity of variance between groups was assessed using the Levene’s test. Where the assumption holds a Students t test was used, otherwise the Welch’s t test was applied. Odds ratios (ORs) were used to express categorical outcomes and patients without DOAC were used as a reference group. IBM SPSS Statistics (version 24.0; IBM Corp. Armonk, New York) for Windows was used for the statistical analyses.

## Results

Of the 314 included patients, 47 patients (15%) were DOAC-users before the hip fracture and 267 patients (85%) were not using anticoagulation before the fracture (Table 1). Hip fracture patients using DOAC were more likely to have a high ASA class (ASA 3–5) compared to non-users.

**Table 1:** Baseline data for the included hip fracture patients in our study (*n* = 337)

	Antithrombotic medication			
	Total	No anticoagulants	DOAC	*p* value
Total *n* (%)	314 (100)	267 (85)	47 (15)	
Mean age (SD)	82.1 (9.2)	81.8 (9.5)	84.2 (7.4)	0.05
Women (%)	221 (70)	190 (71)	31 (66)	0.47
Cognitive impairment (%)	108 (34)	93 (34.8)	15 (31.9)	0.61
ASA class (%)				0.003^a^
ASA 1	8 (2.5)	8 (3.0)	0 (0.0)	
ASA 2	120 (39)	110 (42)	10 (21)	
ASA 3	158 (51)	128 (48)	30 (64)	
ASA 4	27 (8.0)	20 (7.5)	7 (15)	
ASA 5	1 (0.3)	1 (0.4)	0 (0.0)	

^a^Pearson Chi Square test has been used to compare patients in each anticoagulant group with either ASA classes 1–2 or class 3–5. When comparing the rate of cognitive impairment reported between the study groups, patients with unknown preoperative cognitive status were excluded (*n* = 20)

### Time to surgery and hospital stay

DOAC-users and non-anticoagulated patients had similar time interval from admission to surgery (29 vs 26 h, *p* = 0.26, respectively) and similar length of hospital stay (LOS) (6.6 vs 6.1 days, *p* = 0.34, respectively) (Table 2).

**Table 2:** Surgical delay, length of hospital stay, type of anaesthesia, perioperative complications and mortality reported among hip fracture with DOAC or no anticoagulation prior to the fracture (*n* = 314)

Antithrombotic medication				
Hospital stay	Total	No anticoagulants	DOAC	*p* value
Hours from admission to surgery (SD)	26.5 (18.2)	26.1 (19.0)	28.9 (12.9)	0.26
LOS (SD)	6.2 (2.9)	6.1 (2.9)	6.6 (2.2)	0.34
General anaesthesia (%)	**32 (10%)**	**10 (3.8%)**	**22 (47%)**	**0.001**

Perioperative complications				*p* value
Mean blood loss during surgery (SD)	219 mL (208)	218 mL (209)	223 mL (204)	0.9
Mean fall in haemoglobin (SD)	1.90 (1.30)	1.89 (1.25)	1.95 (1.63)	0.8
Mean SAG transfused per patient (SD)	0.81 (1.16)	0.80 (1.17)	0.85 (1.10)	0.8

				OR (95% CI)
Number of patients transfused (%)	134 (43%)	113 (42%)	21 (45%)	1.10 (0.59–2.01)
Reported wound ooze (%)	**27 (8.6%)**	**15 (5.6%)**	**12 (26%)**	**5.8 (2.49–13.3)**

Mortality				OR (95% CI)
In-hospital mortality	11 (3.5%)	9 (3.4%)	2 (4.3%)	1.27 (0.27–6.09)
30-day mortality	39 (12.4%)	34 (12.7%)	5 (10.6%)	1.23 (0.45–3.31)
6-month mortality	70 (22.3%)	59 (22.1%)	11 (23.4%)	0.93 (0.45–1.94)

Bold values indicate more frequent use of general anaesthesia and higher risk of wound ooze in DOAC-users compared to non-users

### Complications

The mean blood loss during surgery for all patients (*n* = 314) was 219 mL. Mean blood loss, fall in haemoglobin and transfusion rates were comparable in both groups (Table 2).

Bleeding complications were reported in three patients (0.9% of all patients); two patients had an excessive bleeding during surgery, while a third patient developed a postoperative haematoma restricted to the operation site. No bleeding complications were reported among the DOAC-users.

Wound oozing with or without bleeding were described in 27 patients (8.6%) and more frequently among DOAC-users than patients without anticoagulation (26% vs 5.6%, respectively) (Table 2). Among all patients (*n* = 314), postoperative wound leakage was associated with a longer hospital stay than for patients without wound exudation (LOS 9 vs 6 days, respectively, *p* < 0.001).

The 30-day mortality for all patients (*n* = 314) was 12%. DOAC-users had corresponding mortality in the hospital, within 30 days and within 6 month compared to non-users (Table 2). Furthermore, 30-day and 6-month risk of readmission were similar between DOAC-users and non-users [30 days: 26% vs 17%, respectively, OR 1.65 (0.80–3.41)] [6 months: 36% vs 26%, OR 1.63 (0.85–3.13)].

### Antiaggregants

Among the DOAC-users, two hip fracture patients were also using clopidogrel (4.3%) while the remaining 45 patients where not using antiaggregant therapy (95.7%).

In the non-anticoagulated group, 92 patients (34.5%) were using 1 antiplatelet drug while ten patients (3.7%) were using two antiplatelet drugs. Time to surgery, perioperative blood loss, transfusion rate, risk of bleeding complications and mortality were similar between non-anticoagulated patients and DOAC-patients both when including and excluding patients with clopidogrel in addition to DOAC.

### Anaesthesia

General anaesthesia was administered to 32 (10%) of all patients. When comparing general to neuraxial anaesthesia, no differences in time from admission to surgery (surgical delay) or LOS was found. A significantly higher percentage of DOAC-users received general anaesthesia than non-users [22 patients (47%) vs 10 (3.8%), *p* < 0.001]. The DOAC-users that received neuraxial anaesthesia (*n* = 25) had significantly longer surgical delay compared to those who received general anaesthesia (35 h vs 22 h, *p* < 0.001). DOAC-users treated with neuraxial anaesthesia trended toward a longer LOS, yet the results were not significant (7.1 vs 6.1 days, *p* = 0.1).

## Discussion

In this single-centre retrospective descriptive study investigating hip fracture patients, the use of DOACs at the time of fracture was not found to influence surgical delay or length of stay compared to non-users. Furthermore, no differences in perioperative blood loss, transfusion rates or risk of bleeding complications between DOAC-users and non-users were disclosed. Hip fracture surgery was more frequently performed in general anaesthesia in DOAC-users, and the use of neuraxial anaesthesia for DOAC-users was associated with a longer surgical delay. This should be seen in relation to primary findings of no difference in surgical delay and length of stay between the compared groups. The high rate of cognitive impairment reported in this study was in line with a previous Norwegian study where 38% of home-dwelling hip fracture patients had cognitive impairment [17].

Studies investigating hip fracture treatment and the use of anticoagulants have so far reported conflicting results. While increased risk of complications was detected in one study [18], other studies discovered no such effect [19, 20]. These diverse findings could be explained by different perioperative administration of anticoagulant drugs. Due to a lack of international established guidelines, patients tend to be treated according to local routines in each hospital.

DOACs are approved for prevention of thromboembolism from non-valvular atrial fibrillation and to treat or prevent recurring deep vein thrombosis and pulmonary embolism [21–23]. These indications may explain why a higher burden of comorbidity was found among hip fracture patients using a DOAC compared to non-users in our study. Despite this increased comorbidity, we were not able to find increased risk of perioperative blood loss, transfusion rates, bleeding complications or mortality for the DOAC-users compared to the less comorbid non-users. Our findings are in contrast to another study reporting a higher one-year mortality among hip fracture patients using DOAC compared to non-users [24]. However, the excess mortality may be explained by higher age, more comorbidity and longer surgical delay than in our patients.

Earlier hip fracture surgery has been associated with reduced LOS and reduced frequency of immobilization-related complications [25–28], and large resources have been applied to promote earlier surgical interventions [29]. Several studies have found increased surgical delay for DOAC-users [16, 18, 24], and the authors question whether the use of DOAC before the hip fracture results in unnecessary long surgical delay [14, 24, 30–32]. In contrast, our DOAC-using patients did not wait significantly longer for surgery than the non-users. Another study investigated hip fracture patients using DOACs compared to matched controls with a median of only 19 h from admission to surgery [30]; no association between surgical delay and perioperative fall in haemoglobin, transfusion rate or reoperation for DOAC-users was found. As our study did not find increased bleeding—and transfusion—complications among patients using DOAC, early surgical interventions appear safe.

The prevalence and risk factors for surgical site infections is sparsely studied in the geriatric hip fracture population even though high age has been identified as a potential risk factor for such infections [33]. Our study revealed wound oozing five times more frequently among DOAC-users than patients without anticoagulation. Still, none of these patients underwent a reoperation due to wound ooze. We need to acknowledge that reoperation due to wound ooze is a late solution to persisting oozing. One earlier study has investigated DOAC-users’ risk of reoperation due to wound ooze and found no relation to surgical delay [30]. On the other hand, when studying hip fracture patients not accounting for chronic anticoagulation, surgical delay has been found to be a risk factor for wound infections [28, 33]. The association between wound ooze and longer LOS found in our study might have implications for health costs and patient treatment following a hip fracture.

In Norway, 80–90% of hip fracture patients are given neuraxial anaesthesia [34], correlating well to the prevalence found in our hospital (90%). There is no international consensus on neuraxial versus general anaesthesia for hip fracture patients [35]. General anaesthesia has earlier been associated with a longer LOS compared to neuraxial anaesthesia [36], yet a meta-study of 400,000 hip fracture patients revealed a clinically insignificant difference of only 0.3 days [37]. The increasing use of DOACs challenge current clinical practice because the potential ramifications of neuraxial anaesthesia in the anticoagulated patient [38]. European guidelines recommend that DOACs should be discontinued before surgery in line with their pharmacokinetic properties [39–42]. Potential neuraxial bleeding can be avoided by giving the hip fracture patients general anaesthesia, possibly explaining why general anaesthesia was used ten times more frequently in patients using DOAC at the time of fracture compared to non-users in our study. One explanation to this finding could be that some DOAC-users were scheduled for delayed surgery to be operated with neuraxial anaesthesia. Another likely explanation is that for these DOAC-users, the chosen modality ended up being neuraxial anaesthesia, because their surgery already had been delayed for other reasons, in example access to theatre and preoperative medical stabilization.

### Strengths and limitations

We studied patients treated at a large trauma hospital using patient records processed by one researcher, thereby increasing the quality and reproducibility of our work. We cannot generalize our findings to other hospitals or countries with other treatment algorithms. However, we believe that our university hospital is representable also for hip fracture treatment in other Norwegian hospitals. Similar surgical delay between DOAC-users and non-users further support comparable preoperative management of all the studied patients.

The sample size was calculated based on our main outcome surgical delay using data from the Norwegian Hip Fracture Register [13]. However, we assessed several other outcomes as well in our study, thereby potentially working with insufficient sample sizes and lack of power. Unfortunately, the size of our study prevented stratified analyses of the different types of DOAC. The retrospective study design allowed us to report associations between DOAC and perioperative outcomes, yet causality cannot be proven. For example, we cannot exclude the risk of confounding by comorbidity when it comes to the choice of anaesthesia and surgical delay. Due to the abovementioned weaknesses of our study, we request future prospective clinical trials targeting hip fracture patients exposed for DOACs and the consequences of fast track surgery versus surgery timed after drug excretion. Further, we acknowledge a need for further studies structurally targeting wound assessment and wound ooze for DOAC-users suffering a hip fracture.

## Conclusion

In our cohort of 314 hip fracture patients DOAC-users did not have increased surgical delay, LOS or risk of reported bleeding complications compared to patients without anticoagulation prior to surgery. Our study does not support delayed surgery for DOAC-users. The increased surgical delay found for DOAC-users operated with neuraxial anaesthesia compared to general anaesthesia should be interpreted with caution.
