# Correction to: Do direct oral anticoagulants (DOACs) cause delayed surgery, longer length of hospital stay, and poorer outcome for hip fracture patients?

**DOI:** 10.1007/s41999-020-00357-4

**Published:** 2020-07-23

**Authors:** Sunniva Leer-Salvesen, Eva Dybvik, Anette H. Ranhoff, Bjørn Liljestrand Husebø, Ola E. Dahl, Lars B. Engesæter, Jan-Erik Gjertsen

**Affiliations:** 1 Department of Clinical Medicine, University of Bergen, Bergen, Norway; 2 The Norwegian Hip Fracture Register, Department of Orthopaedic Surgery, Haukeland University Hospital, Bergen, Norway; 3 Department of Clinical Science, University of Bergen, Bergen, Norway; 4 Department of Chronic Diseases and Aging, Norwegian Institute of Public Health, Oslo, Norway; 5 Diakonhjemmet Hospital, Oslo, Norway; 6 Department of Anaesthesia, Haukeland University Hospital, Bergen, Norway; 7 Innlandet Hospital Trust, Elverum, Norway; 8 Thrombosis Research Institute, London, UK

## Correction to: European Geriatric Medicine 10.1007/s41999-020-00319-w

The original version of this article unfortunately contained a mistake. The presentation of Table 2 was incorrect. The corrected Table 2 is given below. The original article has been corrected.

**Table 2:** Surgical delay, length of hospital stay, type of anaesthesia, perioperative complications and mortality reported among hip fracture with DOAC or no anticoagulation prior to the fracture (*n* = 314)

Antithrombotic medication				
Hospital stay	Total	No anticoagulants	DOAC	*p* value
Hours from admission to surgery (SD)	26.5 (18.2)	26.1 (19.0)	28.9 (12.9)	0.26
LOS (SD)	6.2 (2.9)	6.1 (2.9)	6.6 (2.2)	0.34
General anaesthesia (%)	**32 (10%)**	**10 (3.8%)**	**22 (47%)**	**0.001**

Perioperative complications				*p* value
Mean blood loss during surgery (SD)	219 mL (208)	218 mL (209)	223 mL (204)	0.9
Mean fall in haemoglobin (SD)	1.90 (1.30)	1.89 (1.25)	1.95 (1.63)	0.8
Mean SAG transfused per patient (SD)	0.81 (1.16)	0.80 (1.17)	0.85 (1.10)	0.8

				OR (95% CI)
Number of patients transfused (%)	134 (43%)	113 (42%)	21 (45%)	1.10 (0.59–2.01)
Reported wound ooze (%)	**27 (8.6%)**	**15 (5.6%)**	**12 (26%)**	**5.8 (2.49–13.3)**

Mortality				OR (95% CI)
In-hospital mortality	11 (3.5%)	9 (3.4%)	2 (4.3%)	1.27 (0.27–6.09)
30-day mortality	39 (12.4%)	34 (12.7%)	5 (10.6%)	1.23 (0.45–3.31)
6-month mortality	70 (22.3%)	59 (22.1%)	11 (23.4%)	0.93 (0.45–1.94)

Bold values indicate more frequent use of general anaesthesia and higher risk of wound ooze in DOAC-users compared to non-users

